# Private Ensembles, Public Confidence: A PATE-to-MedPrompt System for Autism Detection

**DOI:** 10.3390/diagnostics16091290

**Published:** 2026-04-25

**Authors:** Alexandru Robert Vlasiu, Marc Eduard Frincu

**Affiliations:** Department of Mathematics and Informatics, West University of Timisoara, 300223 Timisoara, Romania; mfrincu@info.uvt.ro

**Keywords:** mental health, autism spectrum disorder, machine learning, privacy preserving learning, teacher ensemble aggregation, distributed consensus, uncertainty aware diagnosis, clinical decision support

## Abstract

**Background/Objectives:** Early autism screening needs to be both accurate and privacy-preserving, but single-source assessments can miss clinically important context. We therefore study a preliminary integrated framework that combines privacy-preserving questionnaire-based risk estimation with a second reasoning component based on a large language model (LLM) that evaluates symptom narratives. The objective is to test whether structured screening outputs can be translated into uncertainty-aware narrative reasoning within one privacy-conscious workflow. **Methods:** The proposed pipeline links a PATE-style AQ-10 screening stage to a MedPrompt-style consensus reasoning stage that operates on behavioral summaries and transcript-style inputs. Evaluation includes component-wise testing on AQ-10 data, an end-to-end controlled setting, synthetic stress testing, and transcript-only analysis on 26 examples. **Results:** In component-wise evaluation, the combined pipeline reaches ceiling performance on a controlled AQ-10 split, synthetic stress testing reduces accuracy to 97.2%, and transcript-only testing shows that contextual factors such as age substantially improve sensitivity. **Conclusions:** These findings support only a highly preliminary proof-of-concept under constrained evaluation conditions and should be interpreted as motivation for broader external validation rather than as evidence of practical decision-support readiness across settings.

## 1. Introduction

Autism spectrum disorder (ASD) screening is most useful when it happens early, before delays compound across communication, learning, and social development [1,2]. In practice, early decisions are often made under uncertainty, with incomplete information, and within strict privacy constraints. Clinics need workflows that are both clinically responsible and operationally feasible, especially when data are sensitive and distributed across institutions.

Current screening practice faces three connected problems. First, questionnaire-based tools are scalable but can miss contextual details that matter for triage and referral decisions [3,4,5]. Second, richer narrative information from notes or transcripts is harder to standardize and compare across cases. Third, the strongest labeled datasets are often siloed, which limits model development and external validation when raw data cannot be shared safely [6,7].

Compared with our previous standalone studies, this manuscript contributes a preliminary integrated framework that does more than place the two components side by side. Earlier work addressed privacy-preserving questionnaire screening and uncertainty-aware LLM reasoning separately; here we specify the interface between them, describe the uncertainty information passed across that interface, outline fallback and escalation logic for low-confidence cases, and define the reporting structure presented to clinicians during triage.

This paper focuses on that integration gap: how to connect privacy-preserving structured screening with uncertainty-aware narrative reasoning in one coherent decision-support pathway. Our goal is not to automate diagnosis or to claim readiness for clinical deployment. Instead, we propose a clinically grounded architecture that illustrates how structured evidence may be combined with uncertainty-aware narrative processing while preserving confidentiality and making uncertainty explicit.

To address this problem, we propose a two-component pipeline. The first component uses Private Aggregation of Teacher Ensembles (PATE)-style distributed learning over AQ-10 questionnaire data to produce a privacy-protected risk signal and confidence information [6,7]. The second component uses distributed large language model (LLM) consensus (MedPrompt-style prompting with model agreement analysis) to reason over structured case narratives and surface uncertainty for clinician review [8,9,10,11]. In the current manuscript, however, the narrative input in the AQ-10-based scenario is partially synthesized from questionnaire endorsements; accordingly, the second stage should be interpreted as an uncertainty-aware reinterpretation layer rather than a fully independent modality.

The key contributions of this proposal are as follows: (1) a unified end-to-end design that links private questionnaire aggregation to uncertainty-aware reasoning; (2) an explicit interface specification that defines how risk, confidence, and behavioral summaries are transformed into downstream prompts; (3) fallback, escalation, and clinician-facing reporting rules that make uncertainty operational rather than purely descriptive; and (4) an integrated evaluation that includes component-wise testing, end-to-end testing, and a synthetic stress test, without treating the current manuscript as a deployment validation study [6,8].

### 1.1. Summary of Prior Studies and Limitations

Our earlier distributed AQ-10 pre-diagnosis study showed that PATE-style teacher–student learning can preserve privacy while retaining strong screening performance on questionnaire data [6,7]. Its main limitation is scope: it operates on structured tabular inputs and does not define a downstream mechanism for integrating narrative evidence.

Our second study on distributed MedPrompt-style consensus showed that chain-of-thought ensembles and answer shuffling can improve robustness and uncertainty awareness in medical reasoning. MedPrompt refers here to the prompting-and-ensemble strategy introduced for medical question answering rather than to a separate autism-specific model [8,9,10]. Its main limitations are prompt sensitivity and the absence of a privacy-preserving upstream screening interface.

The present manuscript addresses these limitations by combining the two previously separate components into one evaluated workflow. Its novelty is not limited to the handoff step alone: the manuscript also defines the interface variables exchanged between stages, specifies escalation and fallback behavior, introduces a clinician-facing output schema, and evaluates the combined system under both controlled and stress-test conditions. Table 1 summarizes these differences explicitly.

### 1.2. Background and Related Work

This subsection first summarizes our prior studies and their limitations, then details external literature that motivates the present design.

ASD screening tools are valuable for early referral but are not substitutes for formal diagnosis [1,2]. The AQ family of instruments, including brief forms used in practice, enables scalable risk stratification in community and primary-care contexts [12]. Machine-learning studies on questionnaire data report promising performance, yet emphasize tradeoffs between sensitivity and specificity, risks of over-referral, and cohort-dependent generalization [3,4,5,13]. These findings support adding a second reasoning stage to contextualize structured scores rather than relying on one questionnaire signal alone.

Table 2 summarizes the datasets, subjects or cases, and evaluation roles used throughout the study.

PATE introduces a principled approach for private knowledge transfer: teacher models train on disjoint data partitions and transfer consensus to a student via noisy aggregation [7]. In healthcare settings, this design aligns with cross-institution governance constraints because raw records remain local. However, privacy-preserving aggregation can reduce effective signal in low-data settings, so calibration and threshold selection remain central for safe triage deployment [3,4,13].

Chain-of-thought prompting can improve reasoning quality, but output stability remains sensitive to prompt design and exemplar selection [10]. MedPrompt-style methods improve medical benchmark performance by combining few-shot retrieval, rationale generation, and ensemble aggregation across shuffled answer choices [9]. MedQA-style resources are useful for constructing domain-consistent exemplars, though translation from exam-style QA to real clinical narratives remains imperfect [11]. Therefore, uncertainty reporting and disagreement-aware aggregation are necessary for clinical decision support.

## 2. Materials and Methods

This section defines the end-to-end architecture, materials, and methods used to connect privacy-preserving screening with uncertainty-aware LLM consensus. The design is modular, so each component can be validated independently while preserving privacy constraints and auditability [6,7,8].

### 2.1. Architecture Overview

**Figure 1:**
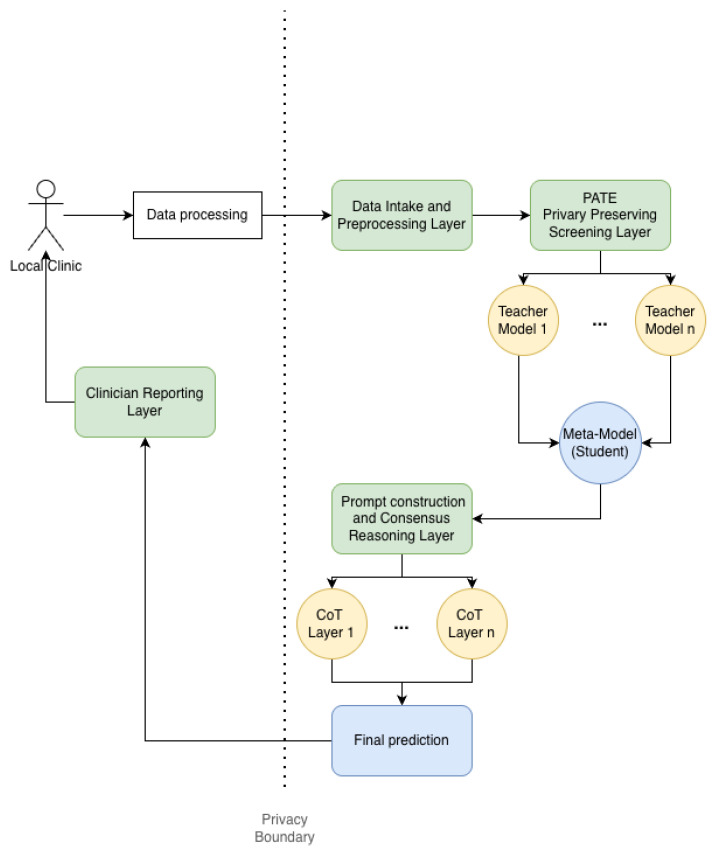
System architecture for privacy preserving screening and uncertainty aware reasoning.

The system is organized into four layers (Figure 1): (1) data intake and preprocessing; (2) privacy preserving screening; (3) prompt construction and consensus reasoning; and (4) clinician facing reporting and logging.

The data intake layer accepts AQ-10 responses and required free text descriptors, standardizes fields, and applies validity checks. The screening layer trains teacher ensembles on partitioned clinic data and distills a student model that outputs a calibrated risk score and confidence interval; this output is treated as an informed opinion rather than a definitive diagnosis, even when the AQ-10 signal appears ideal. The prompt construction layer maps the risk score and the required text into a structured template, injects few shot exemplars, and emits a batch of choice shuffles for ensemble reasoning. The consensus layer aggregates outputs via majority vote and confidence based tie breaks, then produces a final autism detection decision and a short uncertainty aware rationale. The reporting layer logs inputs, outputs, and model metadata for audit and clinician review.

### 2.2. Data Inputs and Preprocessing Layer

The primary structured input is the AQ-10 questionnaire, encoded as binary responses following the standard scoring criteria [3]. In addition, a required free text description summarizes salient behavioral or clinical observations and is used downstream by the reasoning layer. Preprocessing normalizes missing values, enforces valid response ranges, and produces a compact feature vector for the screening model. All raw inputs are retained only within their originating institution; only aggregated teacher outputs and the student model are shared [7].

### 2.3. Privacy Preserving Screening Layer

We adopt a PATE style ensemble of teacher models trained on disjoint partitions of AQ-10 responses [6,7]. Each teacher outputs a class vote and confidence estimate; the aggregated vote is then distilled into a student model to allow deployment without exposing teacher data. The student model outputs (i) a calibrated risk probability, (ii) a confidence or entropy proxy, and (iii) a triage label (e.g., low, moderate, high). Calibration is monitored through held out evaluation and periodic reassessment to prevent drift in risk thresholds [5,13].

In the current prototype, the privacy-preserving aggregation stage follows the RSA-based mechanism introduced in our previous paper rather than a textbook Gaussian- or Laplace-perturbed vote-count implementation. We adopted this modified strategy because, in the AQ-10 setting, direct noise injection on Boolean teacher-vote outcomes produced excessive utility loss relative to the screening objective. Accordingly, the present manuscript should be interpreted as using a PATE-inspired distributed teacher–student design with an RSA-based privacy-preserving aggregation adaptation, not as claiming a standard noisy-count PATE instantiation.

### 2.4. Prompt Construction and Reasoning Interface Layer

Risk scores are translated into structured prompts that separate evidence from inference. A typical template includes: AQ-10 score summary, the PATE screening label as a boolean signal (true or false), and a required behavioral description block. Few shot exemplars are sampled from the MedQA dataset and inserted above the target question to prime reasoning [10,11]. The reasoning task is constrained to a binary autism detection decision (true or false), and answer choices are shuffled to reduce positional bias; each shuffle produces one chain of thought rationale and final answer [9].

### 2.5. Consensus Aggregation and Uncertainty Reporting Layer

The system aggregates *k* shuffled responses using majority vote. If a tie occurs, the system defaults to the PATE screening label and flags the case for clinician review rather than producing a confidence score. We set *k* to 20 ensembles to balance accuracy and latency in distributed inference [8]. The output includes (1) the consensus autism detection decision (TRUE or FALSE), (2) disagreement rate across ensembles, and (3) a short rationale that flags uncertainty and recommends referral when appropriate. Example outputs are: (1) *Decision: TRUE* (autism detected), (2) *Disagreement: 6/20 dissenting votes*, and (3) *Rationale: Reported social communication difficulties and AQ-10 screen positive; refer for specialist assessment*. In the current prototype, a disagreement threshold of more than 30% dissenting votes is used to trigger clinician review; below that threshold, the majority-vote output is reported together with the disagreement rate. This should be interpreted cautiously. The manuscript does not claim that the present rule is optimal for cases in which the upstream PATE output has low confidence or the downstream LLM ensembles disagree strongly. In particular, the tie-break in favor of the PATE label is a pragmatic prototype choice rather than a validated decision rule, and future work should compare it against alternatives such as abstention, forced escalation, or confidence-weighted reconciliation between stages.

### 2.6. Distributed Inference and Performance

Inference is distributed with Ray using 8 physical CPU cores and 0.5 CPU per chain of thought task, enabling up to 16 parallel evaluations [8]. In this manuscript, *acceptable clinical bounds* are defined as turnaround times compatible with pre-consultation triage workflows (on the order of minutes, not hours): specifically, the reported distributed runtime of 359.16 s at 20 ensembles versus over 4800 s sequentially [8]. The distributed design also supports batched processing of multiple patients when clinical workflows require throughput. We do not treat the present MedQA result as a direct benchmark improvement over prior MedPrompt reports, because such a claim would require exact alignment of model version, prompt template, retrieval setup, and MedQA split.

### 2.7. Implementation Notes and Auditability

Every inference run stores a metadata record with prompt version, model version, ensemble size, and timestamp. This enables retrospective audits and model monitoring. Sensitive inputs remain local to their origin; only aggregated results and summaries are stored centrally. The clinician facing report is designed to be interpretable, with explicit uncertainty and a clear separation between screening evidence and LLM reasoning.

### 2.8. Evaluation Plan

To improve readability, evaluation is mapped directly to the pipeline defined earlier (Section 2.1). Stage 1 evaluates the privacy-preserving screening component (Section 2.3) on AQ-10 validation splits from Section 2.12, with accuracy, sensitivity, specificity, and calibration error as primary metrics [5,13]. Stage 2 evaluates the reasoning and consensus components (Section 2.4 and Section 2.5) on MedQA-style prompts from Section 2.12, reporting consensus accuracy, disagreement rate, output stability, and the frequency with which the preliminary disagreement threshold triggers clinician-review escalation [9]. Stage 3 evaluates end-to-end behavior by combining both components in case simulations and measuring referral agreement, uncertainty reporting quality, and mean latency per case, using the distributed constraints defined in Section 2.6. This staged design lets the reader compare isolated component performance with integrated system behavior while checking that uncertainty is exposed rather than hidden.

### 2.9. Datasets

#### 2.9.1. AQ-10 Questionnaire (Screening Input)

We use the AQ-10 child version as the structured screening input. Each AQ-10 item is originally answered as *Yes*/*No*, then converted to binary for model input using the AQ-10 key: responses indicating an autistic trait are encoded as 1, and responses not indicating that trait are encoded as 0 [3]. Table 3 shows a small example of this Yes/No-to-binary encoding used for PATE training.

1.  S/he often notices small sounds when others do not.
2.  S/he usually concentrates more on the whole picture, rather than the small details.
3.  In a social group, s/he can easily keep track of several different people’s conversations.
4.  S/he finds it easy to go back and forth between different activities.
5.  S/he doesn’t know how to keep a conversation going with his/her peers.
6.  S/he is good at social chit-chat.
7.  When s/he is read a story, s/he finds it difficult to work out the character’s intentions or feelings.
8.  When s/he was in preschool, s/he used to enjoy playing games involving pretending with other children.
9.  S/he finds it easy to work out what someone is thinking or feeling just by looking at their face.
10. S/he finds it hard to make new friends.

#### 2.9.2. PATE Training Data

PATE training uses 900 records undersampled from the public AQ-10 dataset by Uppuluri Madhuri [14]. The data are tabular, with one row per respondent and ten AQ-10 feature columns (A1–A10), plus a class label indicating screening outcome (ASD vs. non-ASD). We keep the features strictly binary to align with the PATE teacher classifiers and to simplify the privacy accounting. The subset is stratified to preserve the original class balance before under-sampling. The dataset is used only to train the private teachers and aggregator. No raw responses are passed to the LLM component; the downstream prompt receives the aggregated PATE label together with a short natural-language summary (cf. Section 2.12).

#### 2.9.3. MedQA Examples Used in Reasoning

For the MedPrompt layer, few-shot exemplars are drawn from MedQA, a large-scale, open-domain medical question answering dataset constructed from real-world medical exams [11]. MedQA provides multi-choice clinical vignettes that require linking symptoms, patient age, context, and clinical presentation to a diagnosis or best next step. Items typically include a short case description, a multiple-choice list, and a single correct answer, enabling consistent few-shot formatting. We use MedQA items as reasoning anchors to steer the LLM toward clinical-style differential reasoning without exposing any private AQ-10 records. An example item is shown below [11]:

**Question:** A 3-year-old girl is brought to the pediatrician by her parents who are concerned that she is not developing normally. They say she does not talk and avoids eye contact. She prefers to sit and play with blocks by herself rather than engaging with other children. They also note that she will occasionally have violent outbursts in inappropriate situations. She is otherwise healthy. In the office, the patient sits quietly in the corner of the room stacking and unstacking blocks. Examination of the patient shows a well-developed female with no physical abnormalities. Which of the following is the most likely diagnosis in this patient?

**Options:** A. Autism spectrum disorder; B. Cri-du-chat syndrome; C. Fragile X syndrome; D. Oppositional defiant disorder; E. Rett syndrome

**Answer:** Autism spectrum disorder (A)

### 2.10. Ethical Approval

This study was conducted in accordance with the Code of Ethics and University Deontology of the West University of Timisoara (UVT) [15]. Under the institutional framework referenced in the 2024/2025 Code, formal ethics review and informed consent are mandatory for research conducted on human subjects; because the present work used only secondary, publicly available, and anonymized datasets together with privacy-preserving aggregated outputs, it was treated as exempt from full committee review under the institutional definition of human-subject research. For transcript-based analyses, data usage followed the source repository access conditions (TalkBank/ASDBank) and was restricted to research reporting [6,8,16].

### 2.11. Tools

The implementation uses Python-based components for private learning and distributed inference, including PATE-style teacher–student aggregation and Ray-enabled parallel consensus execution [6,7,8]. In the present prototype description, PATE student inference is assumed to run on the host computer that receives the AQ-10 responses, so raw questionnaire answers need not be sent to teacher nodes during inference; if a broader deployment required server-side coordination, the intended design would be to transmit only downstream results or summaries rather than raw AQ-10 responses. For reasoning, MedPrompt-style prompting is applied with MedQA few-shot exemplars and shuffled-choice ensembles [9,11]. In this manuscript, MedQA is used only as a source of few-shot exemplar style for MedPrompt-like prompting rather than as the evaluation benchmark itself. The prototype reasoning runs use OpenAI’s GPT-5.2 model; the corresponding chat alias is *gpt-5.2-chat-latest*. In the current configuration, the reasoning prompts are sent to an external API rather than processed by a locally hosted open-weight model, so the privacy scope of the LLM stage is weaker than that of the questionnaire aggregation stage. We therefore treat the current system as a prototype threat model only, and note that a deployment-grade version would ideally use either a strong locally hosted LLM or an institutionally governed secure inference environment. Performance reporting uses standard screening and computational metrics (accuracy, sensitivity, specificity, calibration-oriented checks, disagreement, and latency).

### 2.12. Scenarios

We evaluate the pipeline in three components. First, we test the PATE layer alone using a held-out subsample of the 900 training records to measure screening performance under privacy constraints. Second, we test the LLM layer alone on Kaggle-derived AQ-10 profiles rewritten as short clinical narratives, while using MedQA-style few-shot exemplars only to structure prompting and consensus behavior. Third, we run the full end-to-end flow by coupling the PATE Boolean output with a natural-language behavioral profile synthesized from AQ-10 endorsements and then applying MedPrompt consensus.

Example text for a child who endorsed three AQ-10 items (A1, A5, A7):

*AQ-10 endorsements indicate that the child often notices small sounds when others do not (A1), does not know how to keep a conversation going with peers (A5), and finds it difficult to work out a character’s intentions or feelings when being read a story (A7).*

This descriptive text is paired with the PATE output Boolean and used as input to the MedPrompt consensus component. Because the narrative summary is derived from AQ-10 responses in this setting, the integrated experiment should be interpreted as a test of structured-to-narrative evidence translation rather than as fusion of two fully independent modalities.

To broaden evaluation beyond AQ-10, we also describe the role of TalkBank corpora as a complementary, public-language resource. The English Nadig corpus within ASDBank provides parent–child interaction transcripts and metadata, with documentation of collection procedures and transcript conventions [16]. These transcripts are used only as held-out test data in a fallback module when PATE cannot return a boolean label because practitioners have no AQ-10 responses; instead, they provide a short free-text observation, and we generate that text by summarizing interaction patterns observed in the Nadig transcripts. Bang and Nadig (2015) analyze 10-minute mother–child free-play sessions and quantify input features such as word tokens and types, lexical diversity, mean length of utterances (MLU), and number of utterances, reporting no significant group differences in these input measures between matched ASD and typically developing (TYP) groups and finding that input MLU predicts later vocabulary after controlling for initial language level [17]. We do not train on or release TalkBank transcripts; instead, we use high-level linguistic phenomena described in the corpus documentation to ensure that generated summaries remain realistic and clinically plausible.

Example text derived from the Nadig transcript snippet (summarized interactional behavior only):

*During play with toys, the mother offers several prompts to pretend play (phone, baby bottle, tea set) and asks repeated questions. The child gives brief responses, often declines the suggested activities, shifts to blocks, and expresses preferences (e.g., wanting tea first, not liking baby items, noting the phone is not real). The exchange shows turn-taking with short child utterances and limited elaboration, alongside frequent maternal scaffolding.*

## 3. Results

This section presents a component-wise evaluation: (i) PATE-only screening on AQ-10 tabular inputs, (ii) LLM-only reasoning on Kaggle-derived AQ-10 narratives using MedPrompt-style prompting, (iii) end-to-end PATE-to-MedPrompt inference under controlled conditions, and (iv) TalkBank-based fallback using free-text inputs.

### 3.1. PATE-Only Screening on AQ-10

In the PATE-only stage, observed accuracy is 100%, with sensitivity and specificity both at 100%, consistent with the prior pre-diagnosis study. On this evaluation split, this corresponds to 0 false positives and 0 false negatives. This result should not be interpreted as evidence that privacy noise was absent; rather, under the current placeholder privacy setting, teacher-vote margins appear to have been large enough that noisy aggregation did not flip the final label for most cases. Because such ceiling performance warrants careful interpretation, robustness and overfitting analyses are provided in Section 3.4.

### 3.2. LLM-Only Reasoning on Kaggle-Derived AQ-10 Narratives

The LLM reasoning layer is then evaluated in isolation on 200 Kaggle-derived AQ-10 profiles transformed into short clinical narratives. The objective is to quantify reasoning fidelity, answer accuracy, and stability under prompt variation when the PATE component is removed. Few-shot MedQA exemplars are used only to structure the prompt format; they are not the test set. In this prototype configuration, the reasoning runs use OpenAI’s GPT-5.2 model. The model answered 188 of 200 Kaggle-derived cases correctly, yielding 94.0% accuracy and a 6.0% error rate. The standard error is 1.68%, and the 95% Wilson confidence interval is 89.8–96.5% (computed from n=200, x=188). Because this evaluation is performed on AQ-10 narratives derived from the public Kaggle screening dataset rather than on the MedQA benchmark used in MedPrompt, the result should not be interpreted as a direct benchmark comparison with prior MedPrompt reports.

Table 4 summarizes the aggregate LLM-only accuracy and uncertainty on the Kaggle-derived narrative set.

Table 5 provides the compact correctness breakdown for the Kaggle-derived narrative evaluation.

For the ASD-specific subset, 88 of 100 ASD cases are detected (88.0% sensitivity), while all 100 non-ASD cases are correctly rejected (100.0% specificity), corresponding to 12 false negatives and 0 false positives.

The LLM-only prompts are composed from AQ-10 answer patterns transformed into short clinical narratives. Example case derived directly from an AQ-10 profile:

Case 1 describes a child who does not notice small sounds that others miss and concentrates more on the whole picture. In social groups, the child can easily keep track of several different people’s conversations, and they find it easy to go back and forth between different activities. Regarding communication, the child knows how to keep a conversation going with peers and is good at social chit-chat. When reading or observing others, the child finds it difficult to work out a character’s intentions or feelings and finds it easy to work out what someone is thinking or feeling just by looking at their face. In terms of their developmental history and social life, they did not enjoy playing pretend games with other children in preschool and find it hard to make new friends.

### 3.3. End-to-End PATE-to-MedPrompt Flow

The full pipeline is subsequently evaluated, with a PATE-derived Boolean label combined with a structured behavioral summary and processed through MedPrompt consensus. This experiment is designed to avoid treating AQ-10 as a stand-alone ground truth: questionnaire signals can appear near-perfect in controlled settings while remaining vulnerable to atypical clinical presentations. Using the same 200-item set described in Section 2.12, now augmented with the PATE label, end-to-end accuracy is 100% on this controlled split. As discussed in Section 3.4, this is interpreted as a ceiling result under constrained conditions rather than evidence of error-free deployment behavior. Table 6 summarizes the principal metrics.

With PATE in the loop, the full-flow accuracy reaches 100.0%, corresponding to 0 false positives and 0 false negatives on this balanced 200-item set. As with the PATE-only result, this should be interpreted with caution and in the context of the additional robustness analysis in Section 3.4.

### 3.4. Ceiling-Performance Analysis and Overfitting Assessment

The 100% results arise in tightly controlled evaluation settings where label structure is strongly aligned with AQ-10-derived signal patterns and class balance is enforced. Such conditions can produce near-ceiling behavior even when perfect performance is unlikely under broader deployment variability. We therefore do not interpret the ceiling result as evidence that label leakage, dataset-specific separability, or broader contamination concerns have been definitively ruled out. In particular, the present experiments do not provide an independent out-of-distribution clinical validation set, and the synthetic perturbation study should be understood only as a limited robustness check rather than as a substitute for external validation. To assess whether this pattern primarily reflects overfitting, we conducted a synthetic-data stress test designed to increase variability while preserving clinically plausible response logic.

For this check, we generated synthetic AQ-10-like records by probabilistically perturbing item-level Yes/No response patterns observed in the real data, then re-encoded them with the same binary rule used in Section 2.12. Generation constrained marginal item frequencies and class proportions to remain close to the original dataset, while introducing new combinations of endorsed items that were rare or absent in the base split. We then repeated evaluation on this expanded synthetic-augmented set using the same pipeline configuration.

Under this synthetic expansion, accuracy decreased from 100% to approximately 97.2%, and non-zero errors reappeared. A second limitation is that the Kaggle-hosted AQ-10 dataset description does not specify the reference standard behind the final ASD/non-ASD labels, so 100% performance should be interpreted as perfect agreement with the dataset annotations, not as proof of perfect recovery of the underlying clinical condition. A preliminary inspection suggests that these errors are not spread uniformly across the questionnaire. Instead, they are concentrated in borderline response profiles where endorsements from the AQ-10 social-communication items are mixed with less consistent endorsements on items related to attention switching/preference for routine and imagination/inference-style behavior. In other words, the difficult synthetic cases tend to be those in which the pattern no longer matches the clearer low-risk or high-risk combinations seen in the controlled split. This is important because it suggests that the reappearing errors are associated mainly with ambiguous cross-item combinations rather than with one obviously spurious single-item shortcut.

### 3.5. LLM-Only Reasoning on Nadig Transcripts (Free-Text)

We further evaluate the LLM in a separate setting that does not rely on AQ-10 at all. To do this, we use the ASDBank English Nadig corpus (TalkBank ASD access page) and the accompanying study by Bang and Nadig (2015) as a clinical grounding reference for language development patterns in autism [16,17]. We wanted a test set that is independent of questionnaire signals, so we composed prompts from transcript-derived summaries only, with no AQ-10 inputs present.

Based on the Nadig transcripts, we generated short observational summaries. Example:

In this transcript, the child exhibits fluid and responsive verbal communication, engaging in a continuous back-and-forth exchange with the mother. There is a clear presence of shared focus and joint attention, as seen when the child answers contextual questions about a toy phone and later directly asks for assistance to build a tower. The child demonstrates functional symbolic play by participating in a make-believe tea party, assigning “flavors” to the pretend liquid, and performing the physical actions of drinking and stirring. While the child displays a literal perspective by noting that the toy phone is “not real” because it doesn’t function, this does not impede their ability to engage in other forms of imaginative play.

The child also shows strong emotional regulation and social assertiveness, clearly communicating personal preferences by rejecting “babyish” toys in favor of more complex activities like block stacking. Socially, the child is attuned to the mother’s cues, following her suggestions to move to the floor for better stability and participating in social rituals like “cheers.” Based on these objective observations, the child displays high levels of social reciprocity and communicative intent that are inconsistent with the core deficits of autism.

We started from 25 typical and 13 ASD transcripts, then undersampled to a balanced 13–13 split for the main comparison (26 cases total) so that ASD and non-ASD cases would contribute equally to the reported accuracy. On this balanced transcript set, the baseline prompt achieved 65.4% accuracy (17/26), with sensitivity 84.6% (11/13) and specificity 46.2% (6/13). After adding the child’s age to the prompt, accuracy increased to 88.5% (23/26), while sensitivity remained 84.6% (11/13) and specificity increased to 92.3% (12/13). Because the sample is small, we now report Wilson 95% confidence intervals for sensitivity and specificity and interpret all transcript results cautiously. Under an exact binomial test against 50% accuracy, the baseline result is not statistically distinguishable from chance (two-sided *p* = 0.1686), whereas the age-augmented result is significant (*p* = 0.0001). A paired McNemar comparison between the baseline and age-augmented prompts gives an exact two-sided *p*-value of 0.0312, suggesting that the improvement is associated mainly with corrected baseline errors on this balanced transcript subset. We also reran the analysis on the full 25 typical + 13 ASD set and observed only very small differences in the overall pattern; we nevertheless report the balanced setting in the main text because it allows ASD and non-ASD performance to be compared without the majority class contributing nearly twice as many cases. Table 7 summarizes the balanced-set metric results, and Table 8 reports the associated confidence intervals and significance tests.

## 4. Discussion

This study was designed to move beyond single-source screening and toward a privacy-preserving, robust workflow that does not treat AQ-10 as absolute ground truth. The results clarify why this is necessary: the AQ-10 PATE layer achieves near-ceiling performance in controlled screening, yet transcript-only inference is less reliable without additional context. The Nadig transcript experiments show that narrative-only prompts can miss ASD cases and that clinically simple variables, such as age, can materially change the balance between sensitivity and specificity in this small fallback setting. Collectively, these findings illustrate how a proof-of-concept system might be structured to operate both with and without AQ-10, using privacy-preserving aggregation when questionnaire data are available and LLM reasoning over structured narratives when they are not. However, given the constrained AQ-10 split and the transcript-only analysis on just 26 examples, these results should not be read as validation of practical performance across real clinical settings.

From an implementation perspective, the pipeline supports multiple clinical entry points. When AQ-10 is available, the PATE student supplies a calibrated risk signal and the LLM layer cross-checks this signal against symptom narratives, reducing over-reliance on questionnaire scores alone. When AQ-10 is unavailable, the same LLM layer operates on transcript-derived summaries; in this setting, incorporating age preserves sensitivity while substantially improving specificity, which is operationally important for triage but still requires cautious interpretation given the small sample. Overall, the findings support only the conceptual plausibility of treating privacy-preserving screening and narrative reasoning as complementary checks rather than substitutes; they do not establish deployment-ready decision support.

One concrete future direction is to investigate whether Adaptive Lightweight Enhanced Block (ALEFB)-style design ideas can make parts of the workflow more efficient and more suitable for privacy-preserving local deployment. ALEFB was introduced as a lightweight feature-enhancement design for measurement-oriented fetal ultrasound plane identification, with emphasis on balancing discriminative accuracy and computational efficiency [18]. In the present context, ALEFB is relevant not as a direct replacement for the current architecture, but as inspiration for compact feature-extraction or feature-refinement modules that could be inserted upstream of the reasoning stage. For example, an ALEFB-inspired lightweight block could be used to learn more compact representations of AQ-10 response patterns, transcript-derived linguistic summaries, or multimodal behavioral descriptors before passing structured evidence into either the private screening model or a local downstream classifier. This could be valuable in future autism-screening systems where compute, latency, and deployment privacy constraints make smaller edge-deployable models preferable to large external inference services. A second direction would be to study whether ALEFB-style adaptive blocks can improve robustness on borderline cases by emphasizing cross-item interactions that are currently difficult in the synthetic stress-test setting. These possibilities remain speculative, but they suggest a promising path toward more efficient local models that preserve the paper’s privacy-first motivation while reducing reliance on heavyweight downstream reasoning.

## 5. Conclusions

This work introduces a unified, privacy-first screening framework that combines PATE-protected questionnaire aggregation with LLM reasoning over structured narratives. The principal result is that the integrated architecture functions coherently under controlled evaluation: in the AQ-10 split, both PATE-only and end-to-end performance reached 100% (0 FP, 0 FN), while a synthetic-distribution stress test reduced accuracy to 97.2%, indicating strong but non-perfect generalization under broader variability. In LLM-only evaluation on Kaggle-derived AQ-10 narratives, accuracy was 94.0% (188/200; 95% CI: 89.8–96.5%), supporting the internal consistency of the prompt-consensus procedure on this rewritten screening dataset rather than establishing a direct benchmark improvement over MedPrompt on MedQA. In transcript-only fallback mode, adding age context improved accuracy from 65.4% (17/26) to 88.5% (23/26). Sensitivity remained 84.6% in both settings, while specificity increased from 46.2% to 92.3%. Taken together, these findings support the proposed workflow only as a proof-of-concept on a highly constrained evaluation setup, not as evidence of practical privacy-preserving decision support across settings. The AQ-10 results come from a small controlled split, the transcript-only analysis is limited to 26 transcript examples, and no genuinely independent out-of-distribution clinical validation set is yet included; accordingly, substantially larger, externally validated, and clinically grounded studies are required before any deployment-oriented claims could be justified.

Future work could expand transcript-only evaluation with larger, age-stratified cohorts and clinician-reviewed labels, report confidence intervals and paired significance tests routinely, and study how additional metadata (e.g., language level, developmental milestones) affects sensitivity without sacrificing specificity. Additional directions could include formal calibration analysis across components, integrating uncertainty reporting into clinician-facing outputs, and multi-site validation to quantify robustness under domain shift and varying data quality.

## Figures and Tables

**Table 1:** Comparison of the two prior studies and the present manuscript.

Aspect	Prior AQ-10 Privacy Paper [6]	Prior MedPrompt Consensus Paper [8]	Present Manuscript
Primary objective	Private ASD risk prediction from AQ-10	Distributed LLM consensus for medical reasoning	Integrated screening-to-reasoning workflow
Core input modality	Structured AQ-10 responses	Medical QA items/structured narratives	AQ-10 outputs plus behavioral summaries
Privacy scope	Private questionnaire aggregation only	No private upstream screening stage	Private screening with downstream privacy-scope clarification
Uncertainty handling	Confidence estimated in the screening stage	Disagreement measured in the reasoning stage	Uncertainty passed across stages with review triggers
System interface definition	Student-model output only	Prompt-and-ensemble setup only	Explicit transfer of risk, confidence, and symptom summary
Clinical workflow specification	Standalone screening model	Standalone reasoning module	Unified triage workflow and reporting format
Evaluation scope	AQ-10 screening only	Reasoning only	Component, end-to-end, and stress-test evaluation
Main limitation	No narrative integration	No private screening or ASD-specific integration path	Still preliminary; needs external validation

**Table 2:** Summary of datasets, subjects/cases, and evaluation roles used in this study.

Source	Unit	Count/Subset	Use in Study
Kaggle AQ-10 dataset	Questionnaire records	900 training; 200 balanced evaluation; 200 narrative-derived cases	PATE training and evaluation, plus LLM-only narrative testing
ASDBank English Nadig corpus	Transcripts/child cases	25 typical and 13 ASD available; balanced 13 typical and 13 ASD subset for main comparison	Transcript-only fallback evaluation and case-summary generation
MedQA	Few-shot exemplar items	Exemplar set only; not used as an evaluation set	Prompt-format and reasoning-style exemplars for MedPrompt-like prompting

**Table 3:** Example AQ-10 responses encoded as binary features.

ID	A1	A2	A3	A4	A5	A6	A7	A8	A9	A10
S1	1	0	0	1	1	0	1	0	0	1
S2	0	1	1	0	0	1	0	1	1	0
S3	1	0	1	1	1	0	0	0	0	1

**Table 4:** LLM-only performance on Kaggle-derived AQ-10 narratives.

Model/Prompting	Accuracy	SE	95% CI
GPT-5.2, MedPrompt-style (k=20)	94.0% (188/200)	1.68%	89.8–96.5%

**Table 5:** LLM-only confusion summary on Kaggle-derived AQ-10 narratives.

Outcome	Count	Percent
Correct	188	94.0%
Incorrect	12	6.0%

**Table 6:** End-to-end system performance (PATE + MedPrompt).

Metric	Accuracy	Sensitivity/Specificity	Notes
Full flow	100.0%	100.0%/100.0%	agreement (κ) = 1.00; robustness = 100.0%

**Table 7:** Transcript-only performance (no AQ-10).

Setting	Accuracy	Sens.	Spec.
Baseline prompt	65.4% (17/26)	84.6% (11/13)	46.2% (6/13)
Prompt + age	88.5% (23/26)	84.6% (11/13)	92.3% (12/13)

**Table 8:** Transcript-only statistical summary on the balanced Nadig subset.

Metric	Base	+Age
Accuracy	65.4% (17/26)	88.5% (23/26)
Sensitivity	84.6% (11/13)	84.6% (11/13)
Specificity	46.2% (6/13)	92.3% (12/13)
Wilson 95% CI, sensitivity	[0.5776, 0.9567]	[0.5776, 0.9567]
Wilson 95% CI, specificity	[0.2321, 0.7086]	[0.6669, 0.9863]
Binomial test vs. 50%	p=0.1686	p=0.0001
Paired prompt comparison	Value	
McNemar discordant counts	$n_{01}=6$, $n_{10}=0$	
McNemar exact two-sided *p*	0.0312	
McNemar $\chi^{2}$ (Yates)	4.1667	
Asymptotic *p* (Yates)	0.0412	

## Data Availability

The data presented in this study are available on request from the corresponding author due to privacy and ethical restrictions, as the study includes derived case-level materials based on sensitive health-related information and publicly sourced records that were processed under institutional research constraints.

## Abbreviations

The following abbreviations are used in this manuscript:

AQ-10	Autism Spectrum Quotient 10-item
ASD	autism spectrum disorder
FN	false negative
FP	false positive
LLM	large language model
MLU	mean length of utterance
PATE	Private Aggregation of Teacher Ensembles
PR	precision–recall
TN	true negative
TP	true positive
TYP	typically developing
